# Subjective Socioeconomic Status Moderates the Association between Discrimination and Depression in African American Youth

**DOI:** 10.3390/brainsci8040071

**Published:** 2018-04-20

**Authors:** Shervin Assari, Brianna Preiser, Maryam Moghani Lankarani, Cleopatra H. Caldwell

**Affiliations:** 1 Department of Psychiatry, University of Michigan, Ann Arbor, MI 48109, USA; preisebj@umich.edu (B.P.); lankaranii@yahoo.com (M.M.L.); 2 Center for Research on Ethnicity, Culture and Health, School of Public Health, University of Michigan, Ann Arbor, MI 48109, USA; cleoc@umich.edu; 3 Department of Health Behavior and Health Education, University of Michigan School of Public Health, University of Michigan, Ann Arbor, MI 48109, USA

**Keywords:** socioeconomic status (SES), income, financial difficulty, African Americans, blacks, discrimination, depression

## Abstract

**Background:** Most of the literature on the association between socioeconomic status (SES) and health is focused on the protective effects of SES. However, a growing literature suggests that high SES may also operate as a vulnerability factor. **Aims:** Using a national sample of African American youth, this study compared the effects of perceived discrimination on major depressive disorder (MDD) based on SES. **Methods:** The current cross-sectional study included 810 African American youth who participated in the National Survey of American Life-Adolescent supplement. The independent variable was perceived discrimination. Lifetime, 12-month, and 30-day MDD were the dependent variables. Age and gender were covariates. Three SES indicators (subjective SES, income, and poverty index) were moderators. We used logistic regressions for data analysis. **Results:** Perceived discrimination was associated with higher risk of lifetime, 12-month, and 30-day MDD. Interactions were found between subjective SES and perceived discrimination on lifetime, 12-month, and 30-day MDD, suggesting a stronger effect of perceived discrimination in youth with high subjective SES. Objective measures of SES (income and poverty index) did not interact with perceived discrimination on MDD. **Conclusion:** While perceived discrimination is a universally harmful risk factor for MDD, its effect may depend on the SES of the individual. Findings suggest that high subjective SES may operate as a vulnerability factor for African American youth.

## 1. Introduction

Most of the sociology and epidemiology literature on the association between socioeconomic status (SES) and health has focused on the protective effects of SES [1,2,3]. High SES, measured by income and education, has been shown to have strong protective effects in several longitudinal studies including the Health and Retirement Study (HRS) [4], the Americans’ Changing Lives Study [5,6,7,8], the British Cohort Study (BCS) [9], and the Survey of Health, Aging, and Retirement in Europe (SHARE) [8]. According to Mirowsky and Ross, the protective effect of SES on health is “enduring, consistent, and growing” [10]. Link and Phelan also conceptualize low SES as a fundamental cause of poor health [11,12,13,14]. High SES individuals can avoid risk of illness and minimize the consequences of illnesses when they occur [11,12,13,14]. 

While high SES is traditionally regarded as a protective factor against a wide range of health outcomes [11,12,13,14], the health gain from high SES may be diminished for African Americans [15]. Among adults over age 50, using the Health Retirement Survey (HRS), education and income had stronger protective effects against sustained high Body Mass Index (BMI), insomnia, and physical inactivity for Whites compared to African American men and women [16]. In the Americans Changing Lives (ACL) data, high education attainment was a risk factor for an increase in depressive symptoms in the future among African American men, but not any other group [17]. In the National Survey of American Life, high education was a risk factor for suicidal ideation among Caribbean Black females [18]. The protective effects of education and employment on mortality are smaller for African Americans compared to Whites [15,19].

Not only is there a diminished return of SES for African Americans [20,21], but high SES may even function as a vulnerability factor in this population [22,23,24,25,26,27]. Examples of this finding include studies that have shown worse psychological well-being of high SES African Americans compared to their low SES counterparts [15,16,17,18]. For instance, multiple studies using national samples of African Americans have documented an increased risk of major depressive disorder (MDD) and depressive symptoms for high SES African American youth and adults [22,28]. Another study revealed high social costs associated with education attainment for African American youth [25]. Similar results were also reported on the association between high SES and perceived discrimination (PD) in African American youth [27]. However, most of the existing information is limited to adults [17,23,24,28], and there is much left to be learned about the diminished return of SES for African American youth [22,25,27]. We still do not know the exact developmental passes in which high SES becomes a vulnerability factor for African Americans [17,18,22].

There are a few potential explanations for why high SES can operate as a vulnerability factor for African American families. First, the specific coping mechanisms that African Americans use for upward social mobility (to climb the social ladder) include goal-striving stress (GSS) [29,30] and John Henryism [31]. GSS, defined as the discrepancy between aspiration and achievement, weighted by the subjective probability of success and the level of disappointment experienced if goals are not reached [29,30], increases risk of depression [29,30]. Educational success and attainment may be associated with more social, psychological, and physiological costs for African Americans than Whites [24]. GSS seems to be more detrimental to the mental well-being of high SES African American individuals [29], and increases risk of depression and other undesirable health outcomes [29]. Another potential mechanism is John Henryism, which has shown well-established links to depression and depressive symptoms [32]. Finally, high SES may be associated with social isolation, and multiple strains due to jobs and other roles for African American families [33]. African American families who are upwardly mobile may move out of their communities to more White neighborhoods, which increases risk of discrimination [34]. Such a transition may increase high SES African American families’ exposure and vulnerability to discrimination [23]. High SES may also increase parental expectations for their children [35]. It is also known that low SES African Americans are resilient to poverty, and may use system blaming to protect own mental health [36]. For instance, in one study upwardly mobile African Americans and downwardly mobile Whites reported poor mental health [37]. High levels of stress for African Americans who succeed in predominantly White professions as well as their social distance from the rest of their community may increase their risk of poor mental health [33]. Poor African Americans may have developed resilience mechanisms such as flourishing in the presence of adversities [38,39]. They may face a glass ceiling that limits their success [33]. Other scholars have also written about the psychological pain that high SES African Americans experience [33]. This is why economic success enhances the well-being of Whites but not African Americans [40]. This view is in contrast to the declining significance of race [41], as the experience of social and economic mobility is different for African Americans than Whites [33,42].

PD has a well-established negative effect on the mental health of adults [43,44,45,46] and adolescents [47,48,49,50]. For African American youth, discrimination operates as a specific source of stress [50]. In African American adolescents, discrimination reduces self-esteem [49,50] and increases risk of psychiatric disorders [51] and suicidal behaviors [52]. The effects of discrimination as risk factors for depressive symptoms [49,50] and clinical depression [47,48] are also known. Although PD is highly related to depression and other undesirable mental health outcomes, less is known about the role of SES as a vulnerability factor on psychopathology due to PD.

To extend the existing knowledge on the role of high SES as a vulnerability factor for African American youth, we conducted this study to explore whether high SES becomes a vulnerability factor for the effects of PD on MDD. Specifically, we tested objective and subjective SES as potential moderators of the effect of discrimination.

## 2. Materials and Methods

### 2.1. Design

This is a secondary analysis of existing data. Data of this cross-sectional study were borrowed from the National Survey of American Life-Adolescent (NSAL-A) supplement, 2003–2004. NSAL was conducted as a part of the Collaborative Psychiatric Epidemiology Surveys (CPES) and was funded by the National Institute of Mental Health (NIMH) [53,54,55].

### 2.2. Ethics

The study protocol was approved by the University of Michigan (UM) Institutional Review Board (IRB; B03-00004038-R1). Adolescents’ legal guardians provided informed consent. Assent was obtained from adolescents themselves. Each respondent received $50 as financial compensation. Parental consent was obtained from all parents. All adolescent participants provided assent.

### 2.3. NSAL Participants and Sampling Frame

NSAL enrolled 810 African American youth who were sampled in the NSAL-A. NSAL used a national household probability sampling to enroll Blacks. African Americans in the NSAL were enrolled from large cities or other urban and rural areas.

### 2.4. NSAL-A Sampling

As a supplement to the NSAL, the NSAL-A study screened all Black households for eligible youth who were between 13 and 17 years old and were living in the household. Youth were then selected from a random selection table that were based on the sex of the youth. For families with more than one eligible adolescents in the household, two adolescents were enrolled. As a result, the NSAL-A adolescent sample was non-independent. In response, NSAL-A data were weighted to adjust for non-independence in selection probabilities within the households, and households and individuals’ non-response. The weighted data were then post-stratified to represent national estimates based on sex, age, and ethnicity of Black youth in the U.S. [56,57]. The current analysis was, however, limited to African American youth.

### 2.5. Interview

All interviews were performed in English. Of the interviews, 82% were face-to-face. The remaining 18% of interviews were conducted by telephone. Face-to-face interviews used a computer-assisted personal interviews (CAPI) mode. In CAPI, respondents use computers to answer the survey questions. CAPI enhances the quality of the data collection for long and complex surveys [58]. Interviews lasted an average of 100 minutes. The response rate of African Americans in the NSAL-A was 80%.

### 2.6. Measures

The study measured age, ethnicity, gender, SES (subjective SES, income, and poverty index), daily PD, and MDD (lifetime, 12-month, and 30-day).

*Ethnicity.* Youth, race, and ethnicity were identified according to the parents’ ethnicity in the same households. Youth were considered African Americans if their parents had self-identified as African Americans (being Black, but not having any ancestral ties to Caribbean countries).

*Subjective SES.* Participants were asked if they have less than enough, enough, or more than enough money to live. This variable was measured as an ordinal variable, with three levels ((1) less than enough; (2) enough; and (3) more than enough).

*Family Income.* The study collected family income using self-reported data, via interview by the parents. Income was treated as a continuous measure.

*Poverty Index.* The study also measured poverty index (six levels) based on family income, collected during an interview with parents. Higher scores reflected more severe poverty.

*Major Depressive Disorder (MDD).* Outcomes were lifetime, 12-month, and 30-day MDD. These outcomes were measured using the World Mental Health (WHO) Composite International Diagnostic Interview (CIDI), a fully structured diagnostic interview schedule that was originally developed for the WHO project initiated in 2000 [59]. MDD diagnosis was assigned based on criteria in the Diagnostic and Statistical Manual, 4th Edition (DSM-IV). CIDI is applied by trained lay interviewers [60]. Clinical reappraisal studies have shown high concordance between CIDI-based diagnosis of MDD and blinded clinical diagnoses [59,60,61,62]. CIDI-based diagnosis of MDD is valid for African Americans [63,64,65].

*Perceived Discrimination (PD).* The Everyday Discrimination Scale (EDS) measures chronic and routine PD over the past year [66,67]. The EDS is a subtle measure of experience of discrimination, which is well-validated and widely used. The EDS does not prime the respondent to think about race; it is believed to eliminate cues to prejudice prior to responding to the questions [68]. The basis of this approach is that African Americans report more unfair treatment than do Whites [68], suggesting the existence of racial discrimination in the lives of Blacks. The EDS score correlates with measures of institutional racial discrimination, as well as interpersonal prejudice [69,70]. The original EDS measure includes 10 items. However, NSAL-A added three additional items that reflect perceptions of teacher discrimination. As a result, the EDS measure in this study was a 13-item scale. Although the measure was originally developed and normed among adults; exploratory and confirmatory factor analyses have shown high reliability and validity of the measure in Black youth [67,70,71]. The stem question is: “In your day-to-day life, how often have any of the following things happened to you?” Sample items include: “People act as if they think you are dishonest” and “You are followed around in stores.” The measure uses a Likert response scale, which measures frequency of exposure from 1 (“never”) to 6 (“almost every day”). The frequencies of experiencing each event were added to capture overall experience of discriminatory events that occurred within the past year. This approach allows capturing the degree (not the count) of experiencing discrimination as stressors. Higher scores indicated more exposure to discriminatory events in the previous year. PD was treated in this study as a continuous measure (α = 0.86)

### 2.7. Statistical Analysis

This is a secondary analysis of existing data. We analyzed data using Stata 13.0 (Stata Corp., College Station, TX, USA) to accommodate the complex sampling design of the NASL-A. Stata uses Taylor series approximation to estimate variances based on the complex designs. Thus, all inferences reflect the study’s complex design. All percentages reported in this study are weighted and thus representative of the nation’s population. Adjusted odds ratio (OR), their associated 95% confidence intervals (CIs), and *p* values were reported. *p* values less than 0.05 and 0.1 were considered statistically significant for main effects and interaction effects, respectively [72].

Survey logistic regressions were used for multivariable analysis. We ran separate models for lifetime, 12-month, and 30-day MDD as the main outcomes. In all of our models, the main predictor of interest was PD. Age and gender were covariates. Three SES indicators (subjective SES, income, and poverty index) were focal moderators.

First, we ran logistic regressions without interaction terms. These models included one SES indicator, covariates, and PD. We then ran three models with the following SES by PD interactions: (1) subjective SES × PD, (2) income × PD, and (3) poverty index × PD. In all models with the interactions, the main effects were kept in the model, even when they were not significant. We also ran models specifically for males and females.

## 3. Results

Table 1 describes age, SES (centered family income, subjective SES, and poverty index), PD, and MDD (lifetime, 12-month, and 30-day) in the pooled sample of African American youth.

Table 2 reports bivariate correlation between demographic, SES, and MDD. As this table shows, poverty index was associated with income. Subjective SES was associated with poverty index, but not with family income. Lifetime, 12-month, and 30-day MDD were all correlated with each other. PD was positively correlated with lifetime, 12-month, and 30-day MDD. However MDD was not correlated with any of the SES measures.

**Figure 1:**
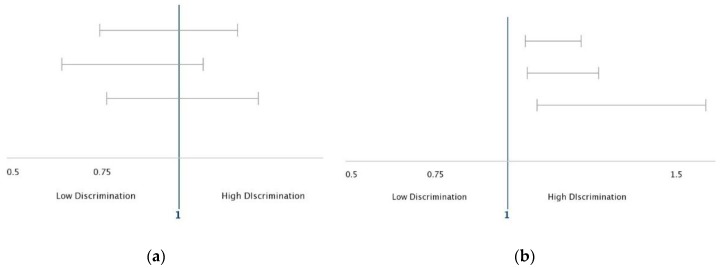
Association between perceived discrimination and depression in low and high SES groups. (**a**) Low SES; (**b**) High SES.

Table 3 summarizes the logistic regressions with MDD (lifetime, 12-month, and 30-day) as the outcome and subjective SES as the moderator. PD was associated with higher risk of lifetime, 12-month, and 30-day MDD in the pooled sample. Subjective SES and gender were not associated with MDD (lifetime, 12-month, and 30-day). PD interacted with subjective SES, indicating a stronger link in the high SES group (Table 3, Figure 1).

Table 4 shows the results of logistic regressions with MDD (lifetime, 12-month, and 30-day) as the outcome and poverty index as the moderator. PD was associated with higher risk of lifetime, 12-month, and 30-day MDD in the pooled sample. Poverty index and gender were not associated with MDD (lifetime, 12-month, and 30-day). PD did not interact with poverty index on MDD (Table 4).

Table 5 presents the results of six logistic regressions. In all of these models, PD was the independent variable, MDD (lifetime, 12-month, and 30-day) was the dependent variable, and family income was the moderator. PD was associated with higher risk of lifetime, 12-month, and 30-day MDD in the pooled sample. Family income and gender were not associated with MDD (lifetime, 12-month, and 30-day). PD did not interact with family income on risk of MDD (Table 5).

## 4. Discussion

While our study showed the effect of discrimination on MDD in the pooled sample of African American youth, it also revealed systemic interactions between subjective SES and discrimination on lifetime, 12-month, and 30-day MDD. This finding suggests that high subjective SES may function as a vulnerability factor and increase African American youth’s vulnerability to the effect of discrimination on MDD.

There are very few studies that suggest high SES may operate as a vulnerability factor for African Americans. Neblett, Bernard, and Banks found high SES (maternal educational attainment) as a vulnerability factor for the link between racial discrimination and psychological distress [26]. Although not conducted with a nationally representative sample, the study by Neblett et al. enrolled an African American teenage sample. The current study extends the results of Neblett’s study in a national sample of African American youth. Hudson et al. found a significant interaction between education level and PD on MDD among African American men. While the interpretation of their findings was that PD reduces the effects of high SES among African American individuals [23], the results could also indicate that high SES is a vulnerability status for the effect of discrimination on MDD. In a study using NSAL-Adults data, PD failed to explain the higher risk of MDD in high income African American men [73].

Comparative to African Americans, there are more studies on high SES as a vulnerability factor for White youth [26,74,75,76,77,78,79,80]. Studies by Luthar and others on youth in suburban affluent communities have found heightened engagement in substance use and internalizing problems (particularly among girls) and sometimes heightened engagement in delinquency (particularly among boys) compared to national norms [81,82,83,84]. These studies evidence the deleterious consequences of high SES for youth. Luthar et al. argued that high SES may increase the vulnerability of families and particularly children [85]. Multiple mechanisms behind vulnerability of upper-class families have been proposed. Authors attributed behavioral problems to the high achievement pressures of high SES families (perfectionistic expectations), isolation, and low closeness between parents and children [85]. High SES families may also be reluctant to seek help and advice for the less visible behavioral and mental health problems due to a variety of reasons such as privacy concerns, embarrassment, or time constraints, leading to upper-class parents feeling more compelled to handle issues independently [86,87]. These studies suggest that both ends of the economic spectrum confer risk for youth [88]. This pattern is very similar to the findings of a recent study on an increase in PD in both ends of the spectrum of SES among African American youth [27]. However, findings in the literature on White adolescent populations may not necessarily mean the same outcomes for African Americans. Much less is known about the effects of high SES as a vulnerability status in African Americans.

Our findings explain why, among African American youth [22] and adults [17,28], high SES is associated with higher risk of MDD and depressive symptoms. For African Americans, high SES as a vulnerability factor seems not to be limited to youth, as it is also found in other age groups [17,28]. It is also not specific to the effect of discrimination on MDD as it extends to a wide range of health outcomes [17,18,22]. A study using the NSAL-Adult sample showed that among male African Americans, having high education credentials was associated with high suicidal ideation [18]. In the ACL study, only for African American men was high education at baseline predictive of an increase in depressive symptoms over the next 25 years. This effect was absent for other race by gender groups [17]. Similarly, Fuller-Rowell and colleagues found a weaker association between educational attainment and health for African American than for White youth [24]. These findings suggest that the diminished health gain among African Americans may be due to the social costs of upward social mobility in African Americans [25]. African Americans may also experience John Henryism or goal-striving stress for their upward social mobility, which come with considerable psychological costs [29,30,31].

These findings extend the existing theories that explain the health effects of SES. Link and Phelan’s Fundamental Cause Theory (FCT; 1995) is mostly focused on the health gain (not psychological costs) associated with high SES. Link and Phelan conceptualize low SES as a fundamental cause of disease [11,12,13,14] across a wide range of health domains [14]. We argue that, due to structural factors, high SES may also have a hidden effect for minorities, as high SES may also increase the psychological costs of discrimination for African Americans. Thus, these findings propose discrimination as a mechanism for African Americans’ diminished return of SES [20,21]. However, discrimination did not explain the higher risk of MDD in high income African American men [89].

Our finding of subjective, but not objective, SES acting as a moderator of PD’s effects on MDD also adds to the existing literature and provides new insight. We do not see these results as a lack of replication of previous findings. Rather, we propose a cognitive model, suggesting that underlying cognitions about SES and “having enough” are influencing PD and MDD.

### 4.1. Limitations

Our findings should be interpreted with limitations in mind. Our first limitation was the study’s cross-sectional design. The findings thus do not reflect causal, but simply associative relationships [90]. Second, our study controlled for some, but not all, important confounders, such as parental education, family structure, and living place. Future research may control for parental education or simply examine the moderating effect of parental educational attainment as a proxy for SES. Research should also replicate these findings using some composite index of SES. In this study, only subjective SES, a measure which contains its own problems, was a moderator of the effect of discrimination. Although subjective SES is a limited measure, it suggests that the path may be cognitive, and related to expectations, not the life conditions due to subjective social class. There is a need to replicate these findings using a longitudinal design and with other confounding variables controlled [91,92,93,94]. In addition, we ran several models to conduct the analysis. This may have made the study prone to Type I Error. We could not adjust for multiple comparisons because of the statistical power and sample size. Despite these limitations, our findings contribute to the literature, as few studies with a national sample of African American youth have conceptualized high SES as a vulnerability factor. Still, due to these limitations, the results should not be overstated. This is particularly important as the same results could not be observed for more objective measures of SES.

### 4.2. Future Research

Future research should explore why subjective, but not objective, SES modified the discrimination-MDD link. Replication of these findings may provide evidence for a cognitive model of SES and discrimination on MDD. This may support the theory that it is not objective experiences, but subjective expectations, that have a role in vulnerability to discrimination. This is in line with the role of racial identity [95,96], attribution [89], and vigilance [97] as vulnerability to discrimination. Thus, future research should also examine whether expectation or vigilance, resilience, or coping can explain the moderating effect of SES on this link.

## 5. Conclusions

Our study findings were suggestive of high subjective SES as a vulnerability factor, as discrimination was a stronger correlate of MDD in individuals who have high subjective SES. The effects of race, SES, gender, and discrimination on mental health outcomes are complex and non-linear. 

## Figures and Tables

**Table 1:** Descriptive statistics (*n* = 810).

Characteristics		
	**Mean (SE)**	**95% CI**
Age	14.95 (0.07)	14.81–15.09
SES (Family income)	0.00 (2.12)	−4.29–4.29
SES (Poverty Index)	3.96 (0.13)	3.70–4.23
Perceived Discrimination (Everyday)	5.06 (0.21)	4.64–5.49
	**% (SE)**	**95% CI**
SES (Poor/Subjective)	12.57 (0.01)	9.71–16.13
MDD Lifetime	6.30 (0.01)	4.75–8.32
MDD 12 Months	4.22 (0.01)	3.07–5.78
MDD 30 Days	1.49 (0.01)	0.76–2.92

Major depressive disorder (MDD), socioeconomic status (SES), confidence interval (CI); Source: National Survey of American Life-Adolescent supplement.

**Table 2:** Correlation matrix (*n* = 810).

Characteristics	1	2	3	4	5	6	7	8	9
1 Gender (Female)	1.00								
2 Age (Years)	−0.02	1.00							
3 Perceived financial difficulty	0.05	0.05	1.00						
4 Poverty Index	−0.01	0.06	−0.13 *	1.00					
5 Family Income (USD1000)	0.00	0.05	−0.08	0.60 *	1.00				
6 Perceived Discrimination (Everyday)	−0.09	0.14 *	0.09	0.08	0.06	1.00			
7 MDD Lifetime	−0.02	0.09	0.03	0.04	0.03	0.12 *	1.00		
8 MDD 12 Months	−0.03	0.10 *	0.04	0.03	0.04	0.11 *	0.84 *	1.00	
9 MDD 30 Days	−0.05	0.06	0.04	0.03	0.05	0.13 *	0.48 *	0.58 *	1.00

* p < 0.05; Major depressive disorder (MDD), Source: National Survey of American Life-Adolescent supplement.

**Table 3:** Summary of logistic regression on the multiplicative effects of perceived discrimination and subjective SES on MDD (*n* = 810).

Characteristics	OR	95% CI	OR	95% CI
	Model 1 (Without Interactions)		Model 2 (With Interactions)	
**MDD Lifetime**				
Gender (Female)	1.18	0.59–2.35	1.18	0.59–2.34
Age	1.29 *	1.02–1.64	1.29 *	1.01–1.65
Subjective SES (Perceived Financial Difficulty)	1.04	0.51–2.12	3.44 #	0.82–14.52
Perceived Discrimination (Everyday)	1.10 *	1.01–1.20	1.14 **	1.05–1.22
Perceived Discrimination × SES	-	-	0.84 #	0.69–1.02
Intercept	0.00 ***	0.00–0.04	0.00 ***	0.00–0.03
**MDD 12 Months**				
Gender (Female)	0.96	0.46–2.00	0.96	0.46–1.98
Age	1.33 **	1.08–1.65	1.34 *	1.07–1.68
Subjective SES (Perceived Financial Difficulty)	1.26	0.49–3.24	8.76 **	1.79–42.77
Perceived Discrimination (Everyday)	1.09 #	0.99–1.20	1.16 **	1.06–1.27
Perceived Discrimination × SES	-	-	0.73 *	0.55–0.98
Intercept	0.00 ***	0.00–0.01	0.00 ***	0.00–0.01
**MDD 30 Days**				
Gender (Female)	0.54	0.15–1.94	0.53	0.15–1.87
Age	1.28	0.84–1.95	1.29	0.84–1.99
Subjective SES (Perceived Financial Difficulty)	1.59	0.36–7.06	23.86 *	1.26–450.13
Perceived Discrimination (Everyday)	1.21 *	1.03–1.43	1.34 **	1.10–1.63
Perceived Discrimination × SES	-	-	0.72 #	0.49–1.06
Intercept	0.00 **	0.00–0.11	0.00 **	0.00–0.09

Major depressive disorder (MDD), socioeconomic status (SES), odds ratio (OR), confidence interval (CI). Source: National Survey of American Life-Adolescent supplement. # *p* < 0.1, * *p* < 0.05, ** *p* < 0.01, *** *p* < 0.001.

**Table 4:** Summary of logistic regressions on the multiplicative effects of perceived discrimination and poverty index on MDD (*n* = 810).

Characteristics	OR	95% CI	OR	95% CI
	Model 1 (Without Interactions)		Model 2 (With Interactions)	
**MDD Lifetime**				
Gender (Female)	1.20	0.60–2.41	1.20	0.60–2.41
Age	1.29 *	1.01–1.65	1.29 *	1.01–1.65
SES (Poverty Index)	1.05	0.86–1.29	1.06	0.68–1.64
Perceived Discrimination (Everyday)	1.10 *	1.02–1.20	1.11	0.90–1.36
Perceived Discrimination × SES	-	-	1.00	0.95–1.05
Intercept	0.00 ***	0.00–0.02	0.00 ***	0.00–0.01
**MDD 12-Months**				
Gender (Female)	0.99	0.48–2.06	0.99	0.48–2.07
Age	1.34 **	1.09–1.65	1.34 **	1.09–1.65
SES (Poverty Index)	1.02	0.83–1.25	1.11	0.75–1.65
Perceived Discrimination (Everyday)	1.10 *	1.00–1.20	1.15	0.94–1.41
Perceived Discrimination × SES	-	-	0.99	0.94–1.04
Intercept	0.00 ***	0.00–0.01	0.00 ***	0.00–0.01
**MDD 30-Days**				
Gender (Female)	0.56	0.15–2.06	0.56	0.15–2.06
Age	1.28	0.85–1.93	1.28	0.85–1.94
SES (Poverty Index)	1.05	0.72–1.52	0.96	0.51–1.79
Perceived Discrimination (Everyday)	1.23 **	1.07–1.41	1.18	0.85–1.64
Perceived Discrimination × SES	-	-	1.01	0.94–1.09
Intercept	0.00 *	0.00–0.13	0.00 **	0.00–0.06

Major depressive disorder (MDD), socioeconomic status (SES), odds ratio (OR), confidence interval (CI). Source: National Survey of American Life-Adolescent supplement. * *p* < 0.05, ** *p* < 0.01, *** *p* < 0.001.

**Table 5:** Summary of logistic regressions on the multiplicative effects of perceived discrimination and family income on MDD (*n* = 810).

Characteristics	OR	95% CI	OR	95% CI
	Model 1 (Without Interactions)		Model 2 (With Interactions)	
**MDD Lifetime**				
Gender (Female)	1.10	0.55–2.23	1.10	0.55–2.23
Age	1.32 *	1.03–1.68	1.32 *	1.04–1.66
Family Income	1.00	1.00–1.01	1.00	0.99–1.01
Perceived Discrimination	1.10 *	1.01–1.19	1.10 *	1.01–1.19
Perceived Discrimination × Family Income	-	-	1.00	1.00–1.00
Intercept	0.00 ***	0.00–0.04	0.00 ***	0.00–0.03
**MDD 12 Months**				
Gender (Female)	1.00	0.48–2.06	1.00	0.48–2.06
Age	1.35 **	1.09–1.66	1.34 **	1.09–1.64
Family Income	1.00	1.00–1.01	1.00	0.99–1.01
Perceived Discrimination	1.10 *	1.00–1.20	1.10 *	1.00–1.20
Perceived Discrimination × Family Income	-	-	1.00	1.00–1.00
Intercept	0.00 ***	0.00–0.01	0.00 ***	0.00–0.01
**MDD 30 Days**				
Gender (Female)	0.57	0.15–2.07	0.55	0.14–2.09
Age	1.28	0.85–1.93	1.26	0.86–1.84
Family Income	1.00	1.00–1.01	1.00	0.99–1.01
Perceived Discrimination	1.23 **	1.07–1.42	1.22 **	1.07–1.41
Perceived Discrimination × Family Income	-	-	1.00	1.00–1.00
Intercept	0.00 **	0.00–0.09	0.00 **	0.00–0.08

Major depressive disorder (MDD), socioeconomic status (SES), odds ratio (OR), confidence interval (CI). Source: National Survey of American Life-Adolescent supplement. * *p* < 0.05, ** *p* < 0.01, *** *p* < 0.001.
